# How useful is a casual BP measurement?

**DOI:** 10.1016/j.clinme.2026.100626

**Published:** 2026-07-17

**Authors:** Philip S. Lewis

**Affiliations:** a Stockport NHS Foundation Trust, Stepping Hill Hospital, Stockport SK2 7JE, United Kingdom; b University of Manchester, Faculty of Biology, Medicine and Health, Manchester M13 9PT, United Kingdom

**Keywords:** Blood pressure measurement, Measurement standards, Cuff size, Blood pressure variability, Home blood pressure measurement, Ambulatory blood pressure measurement, Ward blood pressure measurement, Cardiovascular risk, Casual blood pressure measurement, Measurement error

## Abstract

Blood pressure (BP) varies continuously dependent on numerous personal and external factors as well as on the way it is measured and the methodology used. A casual BP reading may give some clues about a person’s cardiovascular health, but risk can only be determined with repeated careful measurements. If high (≥140/90), systematic measurement following approved guidelines using a validated monitor is necessary, especially if the person is symptomatic and/or BP ≥180/120. A low casual BP (<90/60) should be similarly rechecked, especially if related symptoms are present. Despite the availability of guidelines for measuring BP, there is often poor compliance with these standards leading to less-than-ideal risk assessment. The lack of regulation of what BP home monitors are available to purchase, ignorance about how to measure BP accurately, the use of unvalidated monitors and of incorrect cuff sizes may lead to significant under- or over-assessment of BP with potentially avoidable consequences. Casual BP measurements, especially if elevated, need a systematic non-casual approach to remeasurement and interpretation.

## Graphical Abstract

**Figure:**
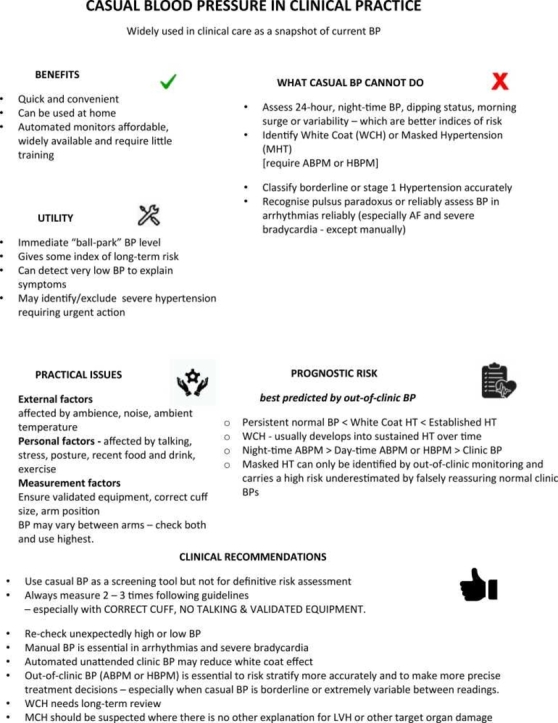

## Key points

• A casual blood pressure (BP) reading provides **only a limited snapshot** of a highly variable physiological parameter.
• Cardiovascular risk correlates more strongly with **long-term average BP, night-time BP** and **home or ambulatory BP** than with a single clinic measurement.
• **White coat** and **masked hypertension** cannot be detected by a casual reading but carry significant prognostic implications.
• Technique errors can distort a single BP reading by **up to 33 mmHg**, reducing its clinical reliability.
• Even a casual BP should follow **standardised measurement protocols** using validated equipment.
• **BP variability** is an independent predictor of cardiovascular morbidity and mortality.

## Introduction

Casual blood pressure (BP) measurement—an opportunistic, single reading taken in clinic, at home or in the community—is one of the most frequently performed assessments in medicine. Although a single BP reading can predict cardiovascular outcomes over long follow-up periods, its predictive value diminishes with time^1^ and improves substantially when combined with long-term average BP.^2^ Because BP is dynamic and measurement error is common, the clinical utility of a casual BP reading requires careful scrutiny.

## BP is a constantly changing physiological variable

Blood pressure fluctuates from beat to beat and is influenced by posture, temperature, stress, pain, bladder fullness, recent activity, talking and numerous other factors. Up to 29 identifiable sources of error can distort systolic BP (SBP) by −23.6 to +33 mmHg and diastolic BP (DBP) by −14 to +23 mmHg.^3^ Numerous other immediate and longer-term factors have been shown to be related to BP (see Table 1).

**Table 1:** Factors affecting BP levels.

**Short-term** • Diurnal variation – often surging in the early morning and dipping at night (but not always) • Autonomic tone • Heart rate and respiration • Posture • Pain, stress, talking • Sleep stage • Exercise • Dehydration • Ambient noise, temperature, music **Longer-term** • Age, sex, birth weight • Body composition (including visceral fat) • Height, arm circumference and length • Genetics • Comorbidities and medications • Diet (sodium, potassium, caffeine, alcohol, supplements) • Physical fitness • Arterial stiffness • Sleep quality and mental wellbeing • Hormonal cycles • Season, atmospheric temperature and pressure • Air and noise pollution • Ultraviolet light exposure • Pet ownership

## Standardised BP measurement: even a casual reading should not be casual

BP should be measured according to recognised standards in clinic, at home or on the ward.

To minimise error, BP should be measured in a resting state:

• seated for 3–5 min
• correct size cuff centred over the brachial artery
• no talking
• back supported
• arm supported at heart level
• feet on floor
• legs uncrossed
• empty bladder
• quiet, comfortable environment.

Three readings should be taken, averaging the last two to allow acclimatisation.^4^ Automated devices that average attended or unattended readings – increasingly used in trials such as SPRINT^5^ and AIM-HY^6^ – reduce white coat effects and improve correlation with outcomes,^7^ but still remain susceptible to physiological variation.

Automated BP monitors may misestimate BP if there is a significant arrhythmia, especially AF and bradycardia, in which case repeated manual readings are preferable, ensuring that cuff deflation is no faster than 3–5 mmHg/s.

### Both arms

Every patient should have BP measured in both arms at least once. A reproducible inter-arm difference should prompt use of the higher-BP arm for future measurements. Inter-arm differences are associated with increased cardiovascular risk.^8^

### Multiple readings

Use a validated monitor and take three readings, averaging the last two or all three. BP typically falls on repeated measurement due to acclimatisation, regression to the mean, and physiological changes in the arm.

### BP measurement is often poor

Inpatient studies show poor compliance with measurement standards^9^:

• wrong cuff size in 37%
• incorrect arm position in 57%
• talking during measurement in 49%.

These errors inflated automated ward BP by ∼10.8/8.85 mmHg compared with correct technique.^10^

## Improving the limited estimate of cardiovascular risk provided by a single BP reading

### Repeat clinic BP on several occasions

If the averaged clinic BP is elevated, repeat measurements on two to four further occasions. Multiple readings reduce random error but still provide only a partial view of long-term BP exposure and resultant risk.

### Home BP monitoring (HBPM)

Home BP correlates more strongly with cardiovascular outcomes than clinic BP,^11^ provided it is performed correctly using:

• a validated monitor
• correct cuff size
• morning and evening readings
• over four to seven days.

HBPM improves diagnostic accuracy, detects masked hypertension, excludes white coat hypertension and provides a more stable estimate of usual BP.

### Ambulatory BP monitoring (ABPM)

ABPM is considered the reference standard for diagnosing hypertension. It provides daytime, night-time, and 24-h averages, identifies dipping patterns, and detects white coat and masked hypertension. A minimum of 14 day-time readings are required. Night-time BP is particularly prognostic; a higher night-time BP or absence of a nocturnal dip indicates increased cardiovascular risk and may be associated with ʻsecondary hypertension’.^12^

## When casual BP measurements do not help

### White coat hypertension

White coat hypertension—elevated clinic BP with normal home or ambulatory BP—is more prevalent in older adults and children and may constitute 9–23% of the population. It is associated with more organ damage, all-cause mortality and cardiovascular events than normotension but less than sustained hypertension.^13^ Importantly, many individuals with white coat hypertension progress to sustained hypertension over a decade. A previously normal out-of-clinic BP cannot be assumed to remain normal; intermittent repeat HBPM or ABPM is required. Treatment decisions should be based on out-of-clinic readings rather than assuming a high clinic BP is benign.

### Masked hypertension

Masked hypertension—normal clinic BP with elevated home or ambulatory BP—carries cardiovascular morbidity and mortality similar to sustained hypertension. Masked uncontrolled hypertension (normal clinic BP but elevated out-of-office BP in treated patients) is particularly dangerous. A casual reading cannot detect masked hypertension, making HBPM or ABPM essential when clinical suspicion is high (e.g., left ventricular hypertrophy, proteinuria, diabetes, chronic kidney disease (CKD), high QRISK score).^14^

### BP variability as an independent risk factor^15^

BP variability, especially long-term visit-to-visit variability, independently predicts cardiovascular events. QRISK3 and QRISK4 incorporate the standard deviation of recent systolic BP readings to account for this.

Key findings:

• Higher visit-to-visit SBP variability increases cardiovascular, stroke, renal risk, and all-cause mortality by 28–46%, independent of mean BP.^16^
• Younger patients and those with lower mean SBP are particularly affected.

A single casual reading cannot capture variability and therefore cannot quantify this component of cardiovascular risk.

## Hidden BP findings

### Arrhythmias

Automated monitors assume regular oscillations in arm volume and often misestimate BP particularly in atrial fibrillation, bigeminy, and bradycardia (<50 bpm). This can lead to low heart rate false alerts. In these cases, manual auscultatory BP is preferable.^17^

### Pulse pressure (PP = SBP – DBP)

• **Narrow PP (<40 mmHg):** seen in tachycardia, sepsis, low cardiac output, tamponade, hypovolaemia, aortic stenosis, postural orthostatic tachycardia syndrome (POTS) and high systemic vascular resistance.
• **Wide PP (>60 mmHg):** seen in aortic regurgitation, arterial stiffness, large ventricular septal defects, patent ductus arteriosus and severe mitral regurgitation.

### Pulsus paradoxus

An inspiratory fall in SBP ≥10 mmHg may be seen in cardiac tamponade, constrictive pericarditis, massive pulmonary embolism, hypovolaemia or severe asthma. It requires deliberate manual assessment and cannot be detected by automated devices.

## Conclusion

A casual BP ≥140/90 mmHg requires formal, repeated assessment using validated equipment and correct technique preferably with confirmatory HBPM or ABPM. Urgent assessment is needed if symptoms include headache, chest pain, breathlessness, visual disturbance, or palpitations, especially if BP ≥180/120 mmHg.

A low casual BP (<90/60 mmHg) may be clinically significant, particularly when accompanied by dizziness, syncope, palpitations, nausea, weakness or confusion. Causes include dehydration, medication effects, pregnancy, cardiac disease and sepsis.

The widespread availability of non-validated home monitors may lead to marked inaccuracies of BP measurement.

Evidence shows that poor measurement is common, even in hospital settings.

There should be nothing casual about BP measurement.

## CRediT authorship contribution statement

**Philip S. Lewis:** Writing – review & editing, Writing – original draft.

## Funding

This work did not receive any specific grant from funding agencies in the public, commercial, or not-for-profit sectors.

## Declaration of Competing Interest

The authors declare the following financial interests/personal relationships which may be considered as potential competing interests: Philip S Lewis reports a relationship with AstraZeneca PLC that includes: consulting or advisory and travel reimbursement. Philip S Lewis reports a relationship with SkyLabs Inc. that includes: consulting or advisory and travel reimbursement.
